# Diclofenac Salts. V. Examples of Polymorphism among Diclofenac Salts with Alkyl-hydroxy Amines Studied by DSC and HSM

**DOI:** 10.3390/pharmaceutics2020136

**Published:** 2010-04-27

**Authors:** Adamo Fini, Cristina Cavallari, Francesca Ospitali

**Affiliations:** 1 Department SMETEC, University of Bologna, Bologna, Italy; 2 Department of Pharmaceutical Sciences, University of Bologna, Bologna, Italy; E-Mail: cristina.cavallari@unibo.it (C.C.); 3 Department of Inorganic and Physical Chemistry, University of Bologna, Bologna, Italy; E-Mail: ospitali@ms.fci.unibo.it (F.O.)

**Keywords:** Diclofenac salts, alkyl-hydroxy amines, polymorphism, DSC/HSM, solubility

## Abstract

Nine diclofenac salts prepared with alkyl-hydroxy amines were analyzed for their properties to form polymorphs by DSC and HSM techniques. Thermograms of the forms prepared from water or acetone are different in most cases, suggesting frequent examples of polymorphism among these salts. Polymorph transition can be better highlighted when analysis is carried out by thermo-microscopy, which in most cases made it possible to observe the processes of melting of the metastable form and re-crystallization of the stable one. Solubility values were qualitatively related to the crystal structure of the salts and the molecular structure of the cation.

## 1. Introduction

A number of papers dealing with the preparation or the structure of some diclofenac salts revealed that some of them precipitate as hydrates when crystallized from water. Thermal and thermogravimetry study or X-ray diffractograms showed that in these salts hydration occurs to different extents, as a function of the counterion, and that also polymorphs are formed. These facts cannot be without consequences when a salt is employed as an active agent in commercial formulations, as is the case of sodium, potassium, diethylamine and pyrrolidine ethanol diclofenac salts. These salts are widely used in oral and topical formulations, and it is important to define the nature of the active agent concerning its molecular weight or the capacity to absorb water and/or to transform as a consequence of its contact with environmental humidity. 

A large series of diclofenac salts with a variety of amines was prepared and tested for solubility [1] and the factors affecting the solubility were examined [2]. However, the interesting behavior of diclofenac/pyrrolidine ethanol salt, which forms polymorphs with different extents of hydration [3,4]; and the easiness of dehydration of some alkaline diclofenac salts [5] suggested better examination of the nature of the solid state of these salts soon after their preparation or after equilibration with water in order to state the formation of hydrates and their stoichiometry or the presence of polymorphs, by suitable techniques. Most salts of this series were in fact previously prepared and examined by means of their melting point using traditional techniques: this parameter, which suffers from subjectivity, often offers uncorrected and partial information on the nature of the solid salt and ignores possible transitions driven by temperature scanning. Therefore thermal analysis in terms of DSC, HSM and TGA furnishes better investigation tools. Moreover, solubility values were previously determined without examining the nature of the solid in equilibrium with the saturated solution. Due to the possibility of changes of the solid state of these diclofenac salts when in contact with water, it can be expected that the samples used to prepare saturated solutions (often obtained from organic solvents) could in many instances be different from those obtained in water. This was found in the case of the pyrrolidine ethanol diclofenac salt; this salt also revealed how complex the behavior of a diclofenac salt can be: the form obtained from organic solvents and that obtained from water are polymorphs and this last form is also bi-hydrate; the first form equilibrates very slowly in water and tends to form relatively stable supersaturated solutions. Since most of the salts were previously prepared in organic solvents to obtain crystalline material, in this paper we prepared a series of these salts in water and, by means of thermal analysis (differential scanning calorimetry - DSC and thermomicroscopy - HSM), assessed the formation of polymorphs, measured the solubility in water and related the solubility to the physical form of the salt present at the equilibrium.

Some years ago we started a project to characterize diclofenac salts prepared with aliphatic amines in order to make a chemical form with high solubility in water available for this potent NSAID and to test their possible new applications [6,7,8]. The behavior in water solution of the salt prepared with pyrrolidine ethanol revealed an interesting aspect that suggested carrying out a systematic examination of the salts prepared with structurally related amines.

Aliphatic bases considered for the preparation of the salts are both linear and cyclic: the parent compound, diethylamine and pyrrolidine respectively for the two series, were chosen as references for an open or cyclic molecular structure. In the present paper we are concerned only with linear aliphatic hydroxy bases. The linear bases in this study carry progressive structural variants, such as increasing number of hydroxy groups (mono-, di- and tri-ethanolamine; TRIS); or combination of ethyl, methyl and hydroxy-ethyl groups (methyl-, dimethyl-, ethyl-, diethyl-monoethanolamine; methyl-, ethyl-diethanolamine). The thermal analysis of these salts was discussed in a previous paper [9]: differences in the thermograms were considered only for few cases. A more systematic examination is carried out in this paper on the differences caused by two different solvents of crystallization. 

## 2. Results and Discussion

### 2.1. Diclofenac salts

The formation of a pharmaceutical salt can modify the physicochemical as well as the biological properties of an ionizable drug, which cannot be predicted from the properties of the parent drug and of the counterion: the final salt in fact represents in most cases a “unicum”, and as a consequence needs a complete pre-formulation study to reveal its peculiarities. The studies carried out on the relationships between the nature of the salt forming agent and the resulting salt cannot be reliable, from this point of view. For instance, increasing hydrophilicity of the counterion can easily be suggested as a simple remedy to increase the solubility in water of the resultant salt and was proposed in the case of erythromycin salts [10]. Also in the case of diclofenac, the need for a soluble salt opened the large field of the aliphatic amines as salt forming agents, different from the usual sodium or potassium hydroxides and, particularly, of those carrying hydroxy groups: with the hypothesis that the higher the hydrophilicity of the counterion, the higher the hydrophilicity of the resulting salt could be [1,3,11,12]. Subsequent research showed that this idea is not completely correct in the case of the diclofenac salts with hydroxy amines [2]. In some cases, diclofenac salts prepared with hydroxy amines proved to be highly soluble in water [11]: however it was demonstrated that, in the case of the pyrrolidine ethanol diclofenac salt, the equilibrium solubility concerns the bi-hydrate polymorph form, the one which is stable in aqueous solution. High solubility values may possibly be found since aqueous solutions of this salt tend to oversaturate and the equilibrium is very slowly reached: in these cases the estimate of the solubility of the metastable forms could be obtained by means of measurement of the intrinsic dissolution rate [13]. Careful investigations on the nature of the solid state [14,4] as well as of the behavior in aqueous solution [6,13,15] of this salt have been described: this was found to depend on the particular structure of the molecule of diclofenac.

### 2.2. The molecule of diclofenac

The molecule of diclofenac, from a point of view of its structure, is a hybrid between a fenamic and an acetic acid class derivative; the characteristic feature is the presence of a secondary amino group (N-H) bridging two aromatic rings and representing the source of a series of intramolecular H-bonds towards a chlorine atom of one and the carboxyl group of the other aromatic ring of the diclofenac molecule. Other H-bonds exist between the carboxyl groups of two different molecules of diclofenac, which face together in a dimer: the dimer form represents a structural unit of the solid state of diclofenac, like that of most carboxylic acids. These aspects originate important consequences. All the H-bonds involve the hydrophilic groups inside the dimer inter- and intra-molecularly and therefore make the diclofenac molecule less available to intermolecular interaction with the environment, such as the water molecules of the solvent. This close association causes the high melting point and the poor solubility in water: the molecule does not form hydrates and only polymorphs have been described, with very limited conformational modification with respect to the more stable structure [16,17].

On the contrary, according to recent literature, diclofenac salts exhibit a variety of hydrates and polymorphs [3]. In particular, the crystal structure of some diclofenac salts prepared with hydroxy amines revealed that these salts exist as ion pairs in the solid state, where the hydroxy groups participate in a network of H-bonds, in some cases together with water molecules of crystallization, that keep anion and cation in a close association: these structural aspects suggested it would be better to define these compounds as complexes rather than simple salts. The complexity of the structures observed made it possible to preview a frequent occurrence of polymorphs among these diclofenac salts as a function of the extent of hydration and/or the nature of the crystallization solvent. It was also reported that in some cases (the salt with 2-amino-2-methyl-1, 3, propanediol) hydration occurs very quickly on contact with water [11]; in some other cases (the salt with pyrrolidine ethanol) hydration is obtained very slowly [15]: both salts can be obtained in the anhydrate form by crystallization from organic solvents. As a consequence, measurement of the solubility in water of diclofenac salts must always be accompanied by a check of the nature of the solid residue in equilibrium with the saturated solution: water, in fact, promotes the formation of hydrates or improves polymorph transition. Similarly, it becomes important to know the experimental conditions where hydrated or de-hydrated forms are stable, as well as the correct molecular weight to be considered, either for dosage or for solubility/structure relationship. 

All these different situations outline the difficulties previously encountered in describing these salts [1], using the determination of a simple melting point. This parameter, in the light of recent results [9], demonstrated that it is impossible to describe the nature of the solid state of these compounds, especially when crystallization of the salts was carried out from different solvents: the melting point, as a parameter of purity of these salts, must therefore also be considered with caution: unforeseen formation of hydrate/solvate/polymorph makes the melting point dependent on the nature of the crystallization solvent.

### 2.3. FT-IR and Raman micro-spectroscopy

**Figure 1:**
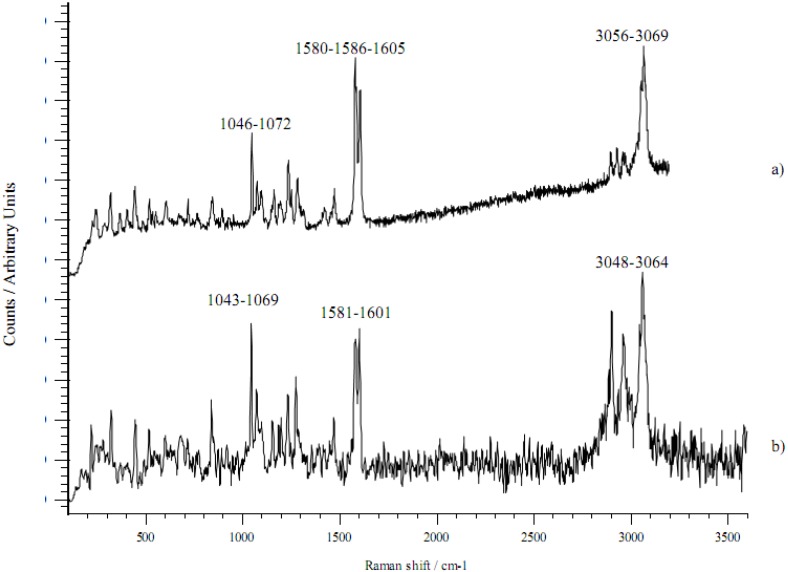
Raman spectra of a) Na diclofenac salt and b) DEA diclofenac salt.

**Figure 2:**
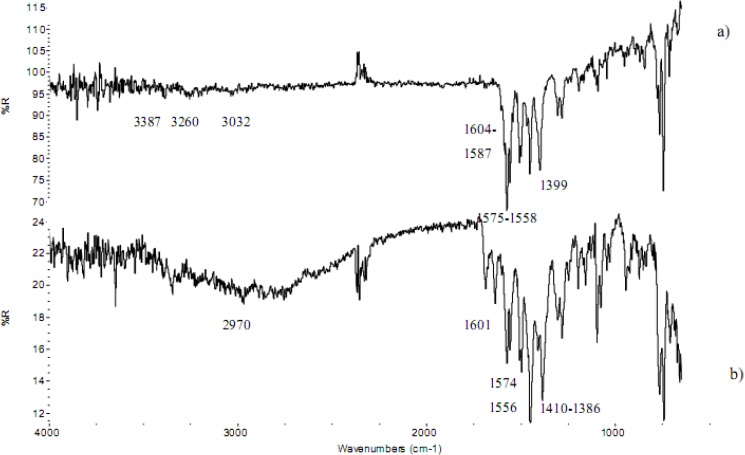
FT-IR (ATR mode) spectra of a) Na diclofenac salt and b) DEA diclofenac salt.

FT-IR and Raman spectra were recorded for all the salts considered here; only those concerning the DEA salt are shown in Figure 1 and Figure 2. Spectra of sodium diclofenac are also reported in order to highlight differences concerning the two cations with differing H-bond capacity.

The Raman spectra of sodium and DEA diclofenac salts are shown in Figure 1. In the Raman spectrum of sodium diclofenac salt the mo st characteristic band is a triplet around 1600 cm^-1^: the bands at 1605 and 1586 cm^-1^ are attributed to ring stretching vibrations of the phenylacetate and dichlorophenyl ring, respectively, and the band at 1580 cm^-1^ is assigned to the asymmetric stretching vibration of the carboxylate. The symmetric stretching vibration of the carboxylate at 1399 cm^-1^ is very weak. The bands at 1072 and 1046 cm^-1^ are characteristic of ring breathing vibrations of the dichlorophenyl and phenylacetate ring, respectively. In the high wavenumber range, the most intense peaks are encountered at 3056 and 3069 cm^-1^, due to aromatic stretching modes. The NH stretching vibration was not detected. 

In the case of the DEA salt, the Raman spectrum shows a red shift (towards lower wavenumbers) and a broadening of the above-mentioned bands: the phenylacetate ring stretching band shifts to 1601 cm^-1^, whereas a single wide band is observed at 1581 cm^-1^. The symmetric stretching of carboxylate (not indicated) shifts at 1385 cm^-1^ and the ring breathing peaks undergo a red shift of 3 cm^-1. In the CH stretching region, the bands shift to 3048 and 3064 cm-1^. The structure of the CH aliphatic stretching bands changes (peak position and intensity) due to the contribution of DEA. The NH stretching mode is not visible.

The changes (peak position shifts and broadening) in the DEA salt spectrum are interpreted by the presence of intermolecular bridge bonds with NH^+^ or OH groups of the hydroxy cation, outlining the formation of an ion pair complex, as reported also by the crystal structures described for these salts (see below).

The micro-FTIR spectra for sodium and DEA diclofenac salts are reported in Figure 2. The sodium diclofenac spectrum also shows two evident and identifying bands characteristic of the carboxylate stretching vibrations at 1575 cm^-1^ (asymmetric) and at 1399 cm^-1 ^ (symmetric). Phenyl rings stretching vibrations are intense in the 1600–1450 cm^-1^ range, particularly at 1604, 1587 and 1558 cm^-1^, whereas ring breathings are attributable at 1077 and 1046 cm^-1^ bands.

The NH and NH-O stretching vibrations are visible at 3387 and 3260 cm^-1^, respectively. These bands, like CH stretching bands, are weaker than expected for a minor response of the ATR technique (employed here) to higher wavenumbers. 

In the DEA diclofenac salt spectrum, changes in peak position of the bands can be seen and are consistent with Raman results. In particular, the phenyl ring stretchings shift to 1601 and 1556 cm^-1^, whereas the band at 1587 cm^-1^ overlaps with the carboxylate asymmetric stretching at 1574 cm^-1^. The carboxylate symmetric stretching shifts to 1386 cm^-1^ and the ring breathings shift to 1069 and 1045 cm^-1^. The NH stretching vibration of the amine group of diclofenac is not visible. At 3650 cm^-1^, the free OH of DEA is visible.

IR spectra were collected also for MEA and TEA diclofenac salts: in these samples, the asymmetric stretching of carboxylate shifts to 1573 (MEA) and 1572 (TEA) cm^-1^.

Therefore, also FT-IR micro-spectroscopy confirmed the shifts of the bands in the hydroxy base salt spectrum with respect to that of the sodium salt spectrum, due to H bridges between anion and cation, as already observed with Raman micro-spectroscopy.

### 2.4. Thermal analysis

The simplest thermogram of a diclofenac salt with aliphatic amines always shows two endotherms: one associated with the melting, the second one with decomposition accompanied by a loss of weight. A fusion between two endotherms is often observed in the temperature range 160–180 ºC, preventing reliable measurements of the parameters associated with each thermal event: these situations were encountered for the salts of this series prepared with MEA, MeMEA and TRIS bases: moreover for the salts prepared with a low boiling amine there is the suspicion that the endotherm recorded at high temperature concerns also the melting of the starting diclofenac (see below).

In some cases, a third endotherm, at temperatures around 100 ºC (such as the case of the salts prepared with MEA), is attributed to de-hydration of the hydrate form of the salt. 

Another type of thermogram additionally contains the complex profile of a polymorph transition: in this case, the thermogram for samples obtained from a single crystallization solvent could offer incomplete description of the compound, since crystallization from an organic solvent or from water could produce different forms, whose thermograms appear very different: water in fact promotes the formation of hydrates and could catalyze the formation of different polymorphs. This last type of thermogram was found [9] for the salt with MEA that exists as a hydrate polymorph. Despite the frequency of polymorphism in this series of compounds, this complex profile could only rarely be observed in DSC thermograms. This could be due to the very close melting temperature of the two forms, but also to the possibility of amorphization that slows down the crystallization process for the diclofenac salts.

Other phenomena concur to complicate the thermal behavior of these salts. It was previously reported [9] that de-hydration in the oven leaves the sample in an (at least partially) amorphous state. In this case, until crystallinity is recovered by long and slow equilibration, ∆H associated with the melting is underestimated and the melting temperature progressively shifts. 

Associating HSM with DSC, each thermal event undergone by the salts on heating can be associated with the right endotherm, when the thermogram profile displays more than one peak. HSM proved useful in investigating the different polymorph forms since re-crystallization of the stable form from the molten mass of the low melting form could be clearly documented by a sequence of photos at the microscope. 

The melting point (m. p.) of the salts was found to be in the range 100–200 °C, EtDEA having a m. p. lower than 100 °C and TRIS a m. p. higher than 209 °C (Table 1). A low m. p. is also displayed by EtMEA (101 °C) and MeDEA (102 °C). In most cases, the melting point of the form obtained from water is lower than that obtained from organic solvent.

### 2.5. Tri-hydroxy cation

The two bases used to prepare diclofenac salts with a tri-hydroxy counterion are very different: the TRIS base being a primary and the TEA a tertiary amine, but with almost similar pKa (7.76 and 8.06, respectively). The TRIS diclofenac salt has the highest melting point among the compounds of this series, and the melting endotherm overlaps that of the decomposition one and displays an irregular shape that prevents reliable thermal parameters being obtained from the thermogram (except peak temperature 209 ºC). The high melting point originates from the structure of the solid state, where a two-dimensional network of hydrogen bonded cations and anions is present, involving the carboxylate group of the anion, the ammonium and hydroxy groups of the cation. A third group of H-bonds between the OH and NH_3_^+^ groups holds together neighboring cations, which could be responsible for the low solubility in water: in fact this close structure contains a dimer between two cations and the solute/solvent interactions do not appear to prevail over the solid state patterns, which are so strongly H-bonded [18].

Also in the case of the TEA salt, the H-bonds keep the anion and cation close together: the O atom of the carboxylate accepts two H-bonds from two hydroxy groups of the cation, while the third OH group provides an H-bond with the same group of another cation, creating an infinite two-dimensional network [19]. A similar pattern of H-bond network was also reported for the same salt of mefenamic acid: the crystal structure of this salt is described as a sequence of cations and anions linked by hydrogen bonds [20]. 

The difference in melting points (ΔTpeak = 71 ºC) clearly outlines how these H-bonded structures operate differently in building up a more compact crystal lattice in the case of diclofenac/TRIS salt. A partial explanation was offered by Castellari and Ottani [19], since these authors reported that, despite the ionic nature of the TEA salt, apparently no direct electrostatic interaction between the positively charged cation and the negatively charged diclofenac anion is observed. These authors attribute this fact to the shielding of the positive centre by the hydroxy ethyl arms of the cation from any intermolecular interaction; additionally an inductive effect due to the O atoms can delocalize the positive charge. The thermograms of these two salts do not change on changing the crystallization solvent from water to acetone, methanol or their mixtures. Whatever the more or less subtle differences between the crystal structures of these two salts, a description of the solid state emerges that agrees with the absence of hydrate forms (the hydroxy groups present on the cation provide all the H-bonds required for a single stable structure of the salt) and the absence of polymorphism (the strong embrace between anion and cation does not leave any freedom for alternative arrangements of the ions or of the hydrogen bonds).

As a result, solubility in water was found to be relatively low in both cases, since to liberate the anion and cation a more complex crystal building must be destroyed than in the case of a simple ionic salt. Solubility is little higher in the case of the TEA salt; this last chemical form of diclofenac was recently proposed [9] as a reference for spectrophotometric determination of diclofenac salts, since it is easy to prepare, shows a simple thermogram and solubility in water (and also in organic solvent) is sufficiently high to prepare stock solution.

The solubility in water of the meclofenamic/TEA salt was found to be almost the same as that of the diclofenac/TEA salt (9.32 *vs.* 7.58 mM), suggesting that a common mechanism operates in limiting solubility in water of these two compounds, whose anions are only slightly different.

### 2.6. Di-hydroxy cation

The structural unit of the DEA diclofenac salt (the form obtained from an organic solvent) has been described as a double-ion pair built up around a pair of cations [21]. The crystal structure of the salt showed an H-bridge between the hydroxy groups of two neighboring cations, by means of a head-to-tail link. This ammonium di-cation generates a two-dimensional network, where each layer forms a sort of sandwich, whose inner part contains all the polar parts: the aromatic rings crowd the surface of the layers and a sequence of 2:2 ion pairs can be taken as descriptive of the solid state of the DEA salt. In this case too, the H-bond network extends over the entire crystal structure (likewise in the case of the TEA salt), but solubility in water appears decisively higher, perhaps for the possibility that unaltered dimer forms enter the dissolution medium. Permanence in solution of the patterns present in the solid state was also hypothesized for other diclofenac salts (e.g., pyrrolidine ethanol diclofenac salt) [1]. Higher water solubility (with respect to the TEA salt) was also reported for the meclofenamic/DEA salt, despite the lower number of hydrophilic groups present in the cation.

**Figure 3:**
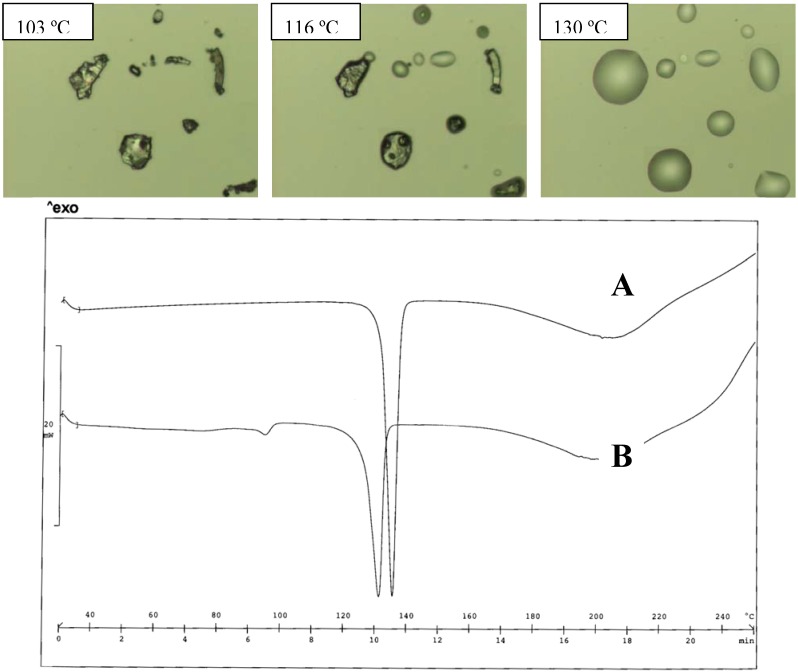
Thermal analysis of the DEA diclofenac salt obtained from acetone (A) and water (B). HSM photos at different temperatures of A (upper panel) and comparison of thermograms for A and B (lower panel).

The DEA salt was found to exist in two different forms as a function of the crystallization solvent: both forms melt at a lower temperature than the TEA salt; the thermograms of the two forms are very simple and do not offer information of formation of hydrates (Figure 3). The form obtained from water has the lowest melting point (120 ºC), but, on heating, no evidence of the polymorph transition could be obtained from the thermogram profile. No information is available on the crystal differences of these two forms, since only the structure of the salt obtained from organic solvent was described [21]. In the case of the meclofenamic/DEA salt, only the form obtained from organic solvent was examined for its crystal structure [20]. Since the proton bonded to the N-atom of the cation is involved in the H-bond network, as reported [21], it is expected that the alkyl substituent causes changes in the solid state of the alkyl-DEA salt, such as MeDEA and EtDEA diclofenac salts: the salts in fact display an even lower melting point than that of the parent compound formed with DEA. 

It appears that just this moiety is responsible for the polymorphism of the DEA salt. The substitution of an H atom with a methyl group prevents the formation of some H-bonds, but does not disturb the crystal packing. The salt melts at a lower temperature, as a consequence of a less close solid state structure, lacking some H-bonds, but the MeDEA salt still appears to exist in only one form: crystallization from water and acetone (as in the cases of salts of this series) does not produce polymorph forms.

**Figure 4:**
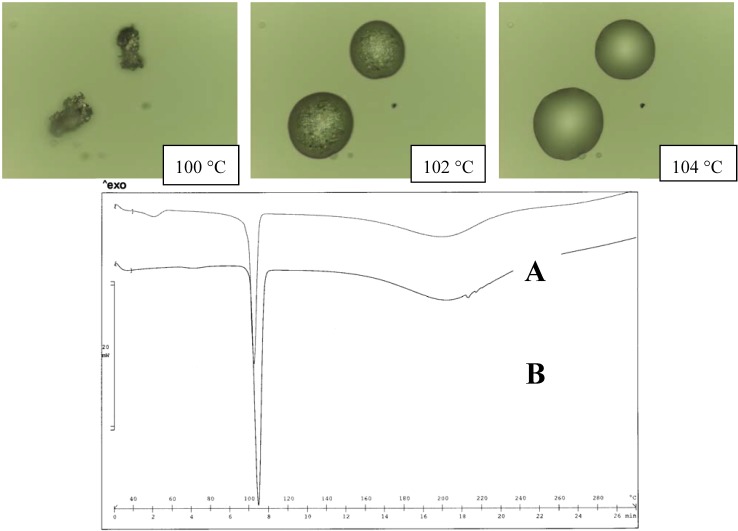
Thermal analysis of the MeDEA diclofenac salt obtained from acetone (A) and water (B). HSM photos at different temperatures of A (top panel) and comparison of thermograms for A and B (bottom panel).

In fact, when the salts were equilibrated in water, as occurs during the determination of the solubility values, the thermograms appear unchanged in the case of the MeDEA derivative (Tpeak 103 ºC). This aspect was also confirmed by the HSM examination of the two forms (Figure 4).

On the contrary, the EtDEA salt, like the DEA one, exists in two forms: the one obtained from water melts at 76 ºC (Tpeak) with a broad and asymmetric endotherm; while the form obtained from acetone melts at 98 ºC: none of these forms are hydrated.

In the case of the EtDEA cation, a longer chain not only disturbs the packing, but introduces the possibility of alternative arrangements of the ethyl substituent: as a consequence, the EtDEA salt can exist in two forms, melting at low temperatures. As in other cases, the form obtained from water melts at very low temperatures (Tpeak 76 ºC) with a large endotherm, suggesting the occurrence of multiple thermal events.

**Figure 5:**
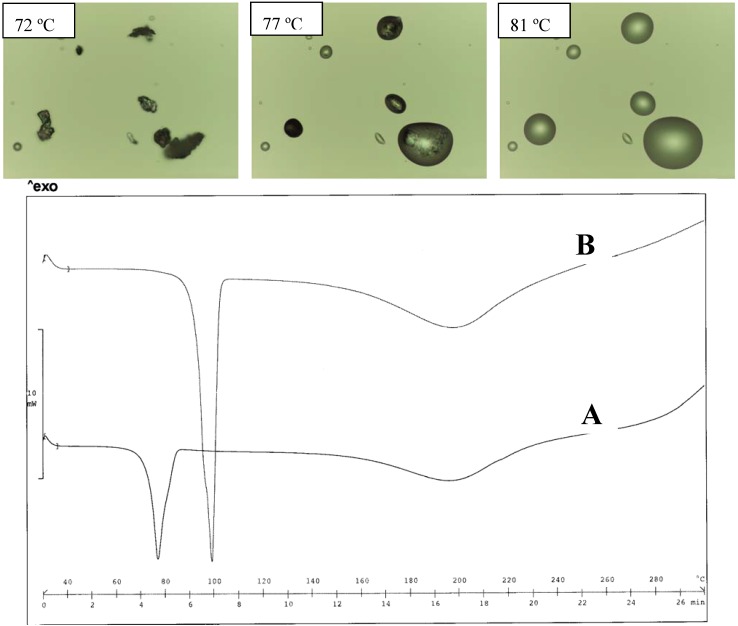
Thermal analysis of the EtDEA diclofenac salt obtained from acetone (A) and water (B). HSM photos at different temperatures of A (top panel) and comparison of thermograms for A and B (bottom panel).

HSM, however, only makes it possible to observe melting, without any possible re-crystallization (even at low scanning rate of the temperature) (Figure 5).

The solubility in water of the ethyl derivative is lower with respect to that of the methyl one, despite a lower melting point: this fact appears due more to the solute solvent interaction (different chain length) rather than to the nature of the solid state and the presence of an additional substituent around the N atom of the base decreases the solubility with respect to the DEA salt.

### 2.7. Mono-hydroxy cation

#### 2.7.1. Mono-alkyl

As previously reported, the MEA diclofenac salt exists as a hydrate polymorph. The crystal structure of this salt is not reported, but the literature offers the crystal structure of MEA salt with meclofenamic and niflumic acids [22,23], whose molecules are structurally related to the diclofenac one. 

**Figure 6:**
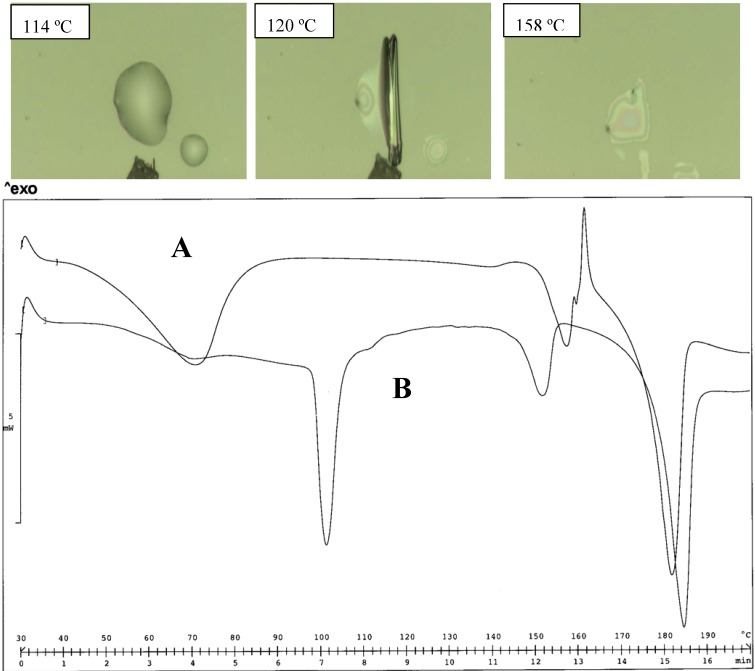
Thermal analysis of the MEA diclofenac salt obtained from acetone (A) and water (B). HSM photos at different temperatures of A, indicating the first melting of the metastable form, re-crystallization and melting of the stable form (top panel), and comparison of thermograms for A and B (bottom panel).

These structures can be taken as representative of the interactions present in the MEA diclofenac salt: the salt exists as an ionic structure, where each anion and cation is involved in multiple H-bonds: the MEA cation, which assumes a gauche conformation, faces the carboxylate group with both the hydrophilic moieties (NH_3_^+^ and OH) and forms a third H-bond with a water molecule; another intramolecular N-H….O H-bond links one carboxylate oxygen with the amino group (Dhanaraj and Vijayan, 1983).

This agrees with the formation of a monohydrate form. The water molecule does not appear to be important in the stability of the crystal structure MEA salt. As a consequence, an anhydrate form of the salt can be obtained either by careful de-hydration or by crystallization by a suitable solvent [22], without altering the original structure. Figure 6 shows the thermograms of the MEA salt as obtained directly from water and after de-hydration. The melting endotherm of the anhydrate form is clearly evident in the sample previously de-hydrated (Tpeak 101 ºC) that undergoes polymorph transition toward the stable form: re-crystallization starts at 120 ºC and terminates at about 140 ºC; a second melting can then be observed at HSM (156 ºC). The TGA profile suggests that a loss of weight, a sign of the occurring decomposition of the salt, starts during the second melting and, at the temperature peak of the melting endotherm, decomposition becomes important. 

The association of DSC, TGA and HSM makes it possible to add a few details to the thermal behavior of the MEA salt previously reported [9], since the endotherm above 100 ºC, previously associated with dehydration, must now, by HSM examination, be also associated with the melting of the metastable form; the small endotherm at higher temperature concerns the melting of the stable form. The last endotherm, which peaks at about 180 ºC, recalls that of the melting of diclofenac acid and was found for other diclofenac salts prepared with volatile amines. It can be hypothesized that during melting, the salt undergoes thermal dissociation, forming both the starting acid and base that evaporates and is responsible for the weight loss, documented by the TGA profile. The remaining diclofenac melts and originates an asymmetric endotherm: this thermal is not documented by HSM. It appears that changing MEA with its alkyl derivatives in the formation of diclofenac salts, an invariant feature is retained, since all the salts considered here were shown to exist in at least two polymorph forms, as demonstrated by DSC and HSM.

**Figure 7:**
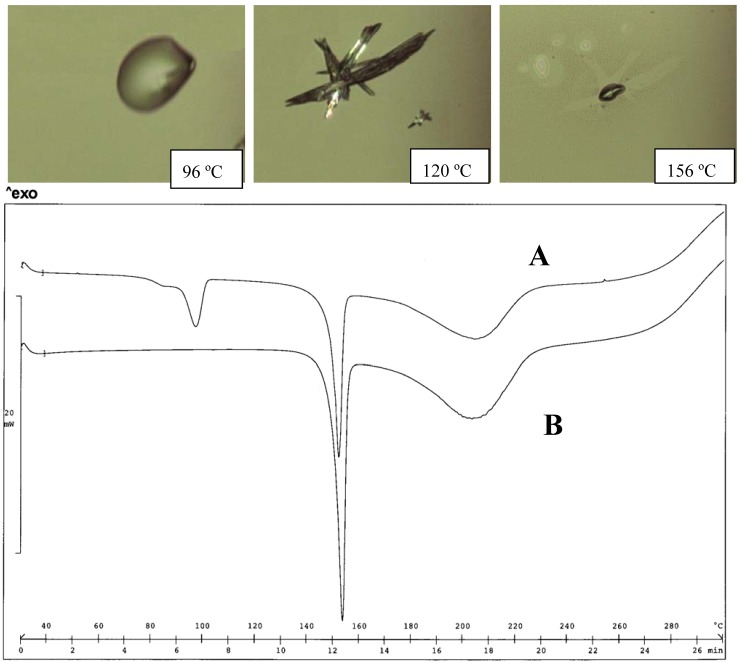
Thermal analysis of the EtMEA diclofenac salt obtained from acetone (A) and water (B). HSM photos at different temperatures of A, indicating the first melting of the metastable form, re-crystallization and melting of the stable form (top panel), and comparison of thermograms for A and B (bottom panel).

The form previously described for the EtMEA salt was that obtained from acetone [9]; a more systematic examination suggested that a metastable polymorph is formed when this salt precipitates from water: this is indicated by an irregular endotherm in the thermogram (Tpeak 97 ºC) and, more clearly, by HSM, able to record the melting of the metastable form and re-crystallization of the stable form. In the HSM photos, the metastable form starts melting at about 95 ºC; above 100 ºC the stable form first crystallizes and then melts at 156 ºC, *i.e.,* at the same temperature as the form obtained from acetone. In this case too, water promotes the formation of a low melting and anhydrate polymorph: however the two forms can be easily obtained by simply changing the crystallization solvent (Figure 7). 

The same situation can be practically encountered with the MeMEA salt: the melting endotherm of the metastable form is irregular when the salt is obtained from (not previously dried) acetone, probably due to the contemporary melting and re-crystallization reactions that could not be separated by DSC. To make this system even more complex, the TGA profile suggests a loss of weight in correspondence with the first melting that continues steadily up to 270 ºC [9]. 

**Figure 8:**
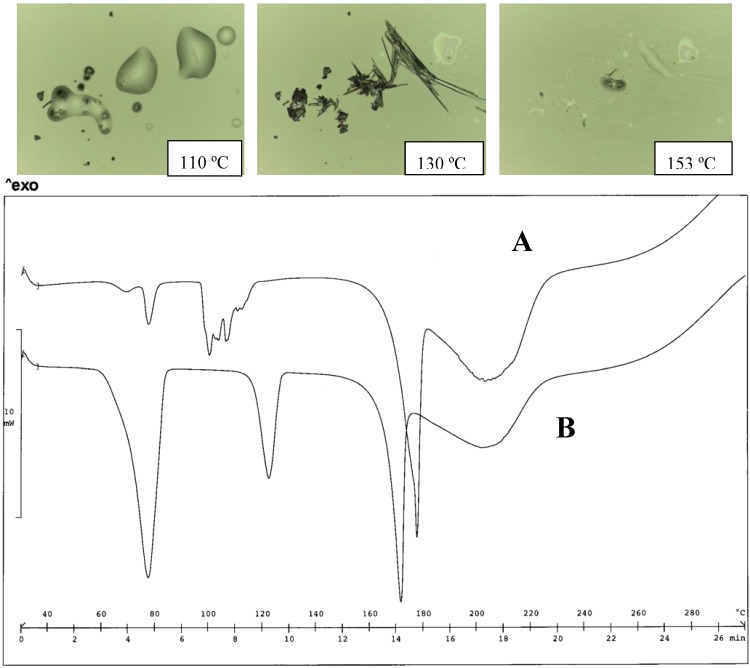
Thermal analysis of the MeMEA diclofenac salt obtained from acetone (A) and water (B). HSM photos at different temperatures of A, indicating the first melting of the metastable form, re-crystallization and melting of the stable form (top panel), and comparison of thermograms for A and B (bottom panel).

The form obtained from water presents a more regular thermogram with three endotherms peaking at 77, 122 and 172 ºC respectively (Figure 8).

The MeMEA salt, like the reference MEA salt, also forms a hydrate polymorph.

The first endotherm is associated with dehydration; the second one represents the melting of the anhydrate form that re-crystallizes soon after, and the stable form starts melting at 160 ºC. Differences can be related to the different experimental treatment of the two samples. Comparison with the thermogram of diclofenac could originate doubts about the attribution of the endotherm at high temperature: HSM photos, however document that it must be attributed to the melting of the salt.

#### 2.7.2. Di-alkyl

**Figure 9:**
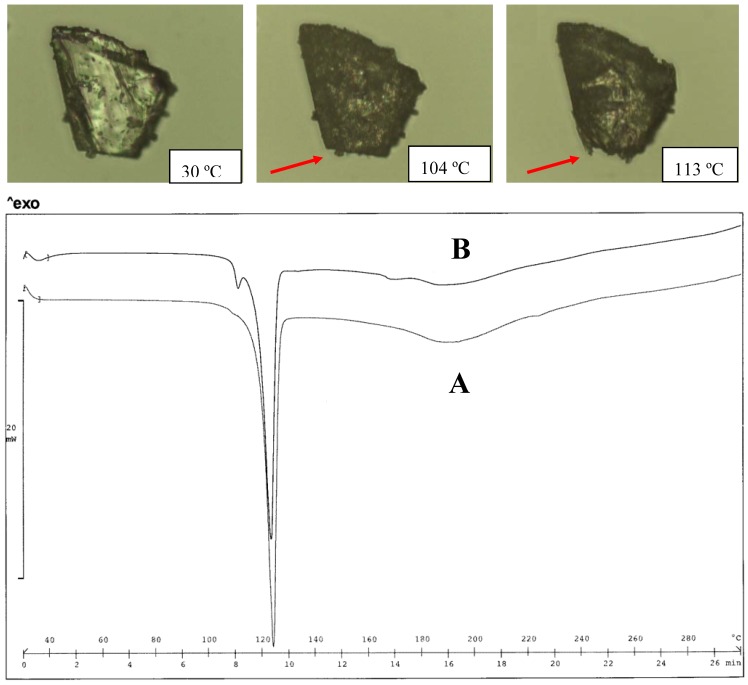
Thermal analysis of the diMeMEA diclofenac salt obtained from acetone (A) and water (B). HSM photos at different temperatures of A, indicating first the darkening of the sample and then little morphological changes, indicated by the arrow (top panel), and comparison of thermograms for A and B (bottom panel).

The presence of two methyl groups carried by the N atom considerably modifies the thermogram of diMeMEA salt with respect to the monomethyl derivative (Figure 9). This salt was previously described [9,11] and it was presumed to exist in two polymorph forms. Two endotherms at relatively low temperature are present in the thermogram: the first one having a lower surface area (at about 109 ºC) and the second one being more regular (Tpeak 123 ºC) that can be associated with the melting of the two forms. 

**Figure 10:**
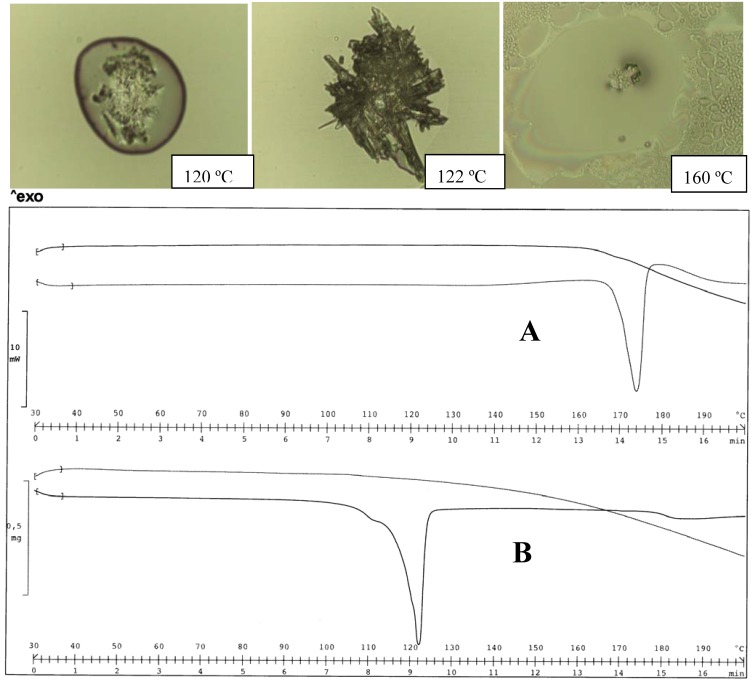
Thermal analysis of the diMeMEA diclofenac salt. HSM photos at different temperatures indicating melting, re-crystallization and final melting of acidic diclofenac, formed by thermal dissociation of the diMeMEA diclofenac salt (top panel). Comparison of thermogravimetric profiles for acidic diclofenac (A) and diMeMEA diclofenac salt (B) (bottom panel).

No sign of a typical polymorph transition could be observed from the thermogram profile, due to the proximity of the two temperature values. HSM showed changes (though not clearly evident) on the crystal surface under examination in the temperature range 112–113 ºC. A re-crystallization was however observed by HSM starting from 123 ºC (Figure 10), *i.e.,* at the peak temperature of the melting of the stable form, but this thermal event is not documented by the thermograms and cannot be associated with the polymorph transition, since this new form melts at about 160 ºC. 

Since a loss of weight is evident in the TG profile (Figure 10, B), starting at the onset of the melting endotherms, from about 100 ºC (KF titration indicates absence of water molecules of crystallization), it can be hypothesized that the new solid form derives from the thermal dissociation of the salt during its melting that liberates the volatile diMeMEA (b.p. 133–135 ºC) to evaporate and leaves acidic diclofenac, which can re-crystallize inside the molten salt vesicles since its m. p. is 173 ºC, and then melt at a higher temperature: the TG profile suggests that also acidic diclofenac decomposes on melting (Figure 10 A ).

The diMeMEA diclofenac salt represents an example of how difficult the correct description of these diclofenac salts with aliphatic amines and their thermal behavior can be: the small endothermic peak could be confused with the presence of an impurity and the loss of weight with dehydration. Only association of different techniques allowed the clarification of the formation of two anhydrate polymorph forms for the diMeMEA diclofenac salt.

**Figure 11:**
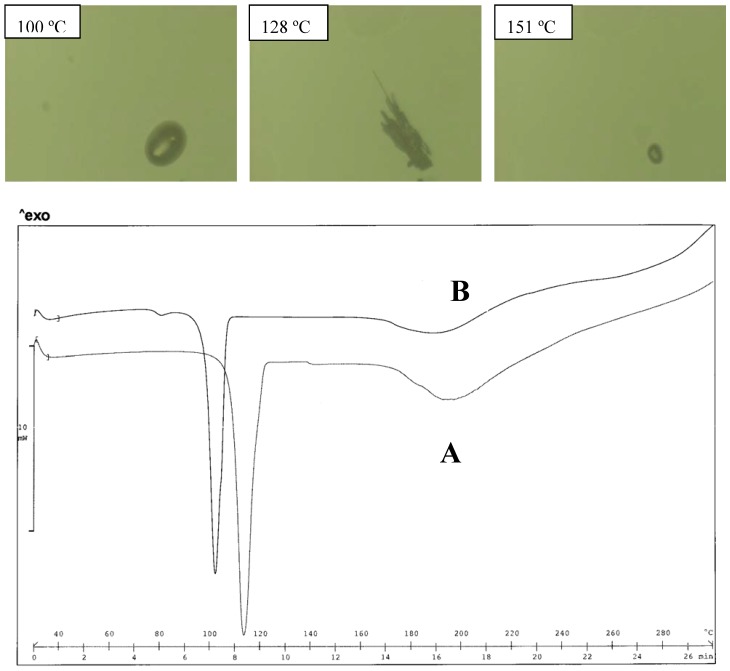
Thermal analysis of the diEtMEA diclofenac salt obtained from acetone (A) and water (B). HSM photos at different temperatures of B, indicating, first the melting of the salt, re-crystallization of the form melting at 151 ºC (top panel); and comparison of thermograms for A and B (bottom panel).

These events can explain the differences between the two endotherms: the heat developed with the crystallization can almost compensate the heat required for the first fusion and the proximity of the two melting temperatures did not allow a thermogram profile expected for a polymorph transition.

The diEtMEA diclofenac salt forms two polymorphs on crystallization from water or from acetone: the two forms have a melting point close together (101 and 112 ºC respectively): there was no evidence of polymorph transition in the thermogram (Figure 11); but in this case too, a weight loss of about 2% can be measured in the temperature range of the melting endotherm, again suggesting thermal instability. HSM analysis documents the occurrence of the decomposition reaction of the salt showing a melting followed by a re-crystallization of a form (128 ºC) that melts at 151 ºC: this situation recalls that described for the diMeMEA diclofenac salt.

### 2.8. Solubility values

On analyzing the solubility values in Table 1 a common aspect emerges for all the compounds examined: solubility of these diclofenac salts with aliphatic amines does not differ too much from the values found for the alkaline salts. Most of them are in the range 20–40 mM and the presence or not of hydroxy groups in the cation does not affect the solubility in water, as could be expected. On the contrary, in some cases it is dramatically depressed by the presence of hydroxy groups, such as that of the TRIS and TEA salts, which display the lowest solubility despite the presence of three hydroxy groups in the cations. Due to internal and strong (as documented by the ΔH values associated with the melting) inter-ion H-bonds, most of the hydrophilicity of the salt proves unfavorable to interact with water and solubility is negatively affected. Moreover, melting ΔS values suggest that the destruction of the crystal lattice of these salts on melting is accompanied by a great loss of order: this aspect can be (even though partially) attenuated, when solubility is considered, by the solvation of anions and cations in an aqueous medium.

Solubility, which is well known to depend on the solid state structure and on the solute/solvent interaction, in this case appears to depend to a higher extent on the first factor. In the case of the TRIS salt this is evident from the highest melting point observed for this group of salts and also from a close network of hydrogen bonds, between anion and cation, as documented by the crystal structure, where the salts present an ion pair; this aspect is also present in the TEA salt, where the longer chains contribute to build up a less compact structure, decreasing the melting point, but not the ΔH of melting, with respect to TRIS salt. This aspect appears more complex for alkyl-hydroxy bases, whose salts have relatively lower melting points, but solubility is of the same order of magnitude.

The solubility can be represented as a complex relationship among the number of substituents on the N atom, the length of their chain, the number of hydroxyl groups and the parameters associated with the solid state of the salt, and for these diclofenac salts it does not appear dependent on only one parameter but rather on a combination of parameters, even though relationships between properties of the salt-forming agents and those of the resulting salts were highlighted for some diclofenac salts with four-carbon primary amines [11].

However, comparing the solubility data for these salts with the melting points of the forms obtained from water it appears that no simple linear relationship can be found for these salts; in the same way no relationship could be observed for the ∆H associated with the melting: in fact, as previously observed, ∆H associated with the melting could be affected by heat evolved during the polymorph transition and therefore ∆H values, as calculated by the parameters of the thermograms, do not reflect only the enthalpy of fusion. As a conclusion, a multi-parameter relationship, recently reported [24] for a number of diclofenac salts, to highlight the factors underlying the solubility in water of these salts, can be considered of limited reliability, since the parameters considered (melting point and ∆H) are in most cases not those concerning forms obtained from water.

**Table 1:** Thermal parameters and solubility in water of the salt forms obtained from water.

	Aliphatic base	Acronym	B. p. (free base) (°C)	MW of the salt (anhydrous)	T_peak_ of the salt melting (°C)	ΔH_m_ (Kcal.mol^-1^)	ΔS _m_(cal.mol^-1^·K^-1^)	Solubility (mM)
1	Mono-ethanolamine	MEA	169	357.23	101*	2.6	7.0	26.5
2	Di-ethanolamine	DEA	269	401.29	130	6.8	16.8	45.0
3	Tri-ethanolamine	TEA	360	445.34	138	15.8	38.4	7.6
4	Tris-methylol aminomethane	TRIS	220	418.15	209	15.3	31.8	3.5
5	Methyl-monoethanolamine	MeMEA	156	372.15	78	1.1	3.1	25.4
6	Dimethyl-monoethanolamine	diMeMEA	135	385.29	111	*	*	54.4
7	Ethyl-monoethanolamine	EtMEA	170	385.29	97	2.7	7.3	33.8
8	Diethyl-monoethanolamine	diEtMEA	161	413.34	113	7.7	19.9	36.0
9	Methyl-diethanolamine	MeDEA	248	415.31	102	7.2	19.2	33.3
10	Ethyl-diethanolamine	EtDEA	246	430.15	76	6.6	18.9	25.2

* see Figure 9: the area surface of the first peak cannot be reliably obtained.

## 3. Experimental Section

### 3.1. Materials

Pharmaceutical grade Diclofenac was a gift (IBSA, Lugano, Switzerland). The following bases: mono-ethanolamine (MEA), di-ethanolamine (DEA), tri-ethanolamine (TEA), tris-methylolamino methane (TRIS); N-ethyl mono-ethanolamine (EtMEA), N,N-diethyl mono-ethanolamine (diEtMEA), N-methyl mono-ethanolamine (MeMEA), N,N-dimethyl mono-ethanolamine (diMeMEA); N-ethyl di-ethanolamine (EtDEA) and N-methyl di-ethanolamine (MeDEA), were commercial samples (Aldrich Italia, Milan, Italy).

Throughout the paper the salts are identified with an acronym of the starting amine.

All the solvents used for crystallization were of pharmaceutical purity grade.

### 3.2. Methods

*Preparation of the salts* – Salts were prepared separately, dissolving equimolar amounts of acidic diclofenac and the appropriate base in acetone and then mixing the two solutions. The salts, according to their solubility in the solvent, either precipitate rapidly, or after cooling at -20 ºC, or after concentration of the final solution, removing excess solvent at room temperature. The products recovered by filtration under reduced pressure were initially dried at ambient conditions for 24 h and examined by DSC. The salts were crystallized both from an organic solvent (acetone or methanol) and from water, and thermograms were compared to show the presence of hydrate or polymorph forms.

*Solubility of the salt* – Equilibrium solubility was determined by adding excess solid to 10 mL distilled water and placing the resulting systems into a water bath thermostated at 25 ºC. After a week, samples of the solution were spectrophotometrically examined at 278 nm. Determinations were carried out in triplicate. Using the molar extinction coefficient for the diclofenac anion, absorbance values were converted into mM values of solubility, which are shown in Table 1.

*Determination of molar extinction coefficient* – Accurately weighed amounts of TEA diclofenac salt were dissolved in water and absorbance of each solution was measured at 278 nm, which represents the maximum in UV diclofenac anion. Plot absorbance *versus* molar concentration is linear in both cases and from the slope the molar extinction coefficient was calculated (ε_M_ = 1.2.10^4^ cm^2^ mole^-1^). This salt was chosen after having found that it does not form hydrates or polymorphs, so the molecular mass can be assessed without problems.

*Differential Scanning Calorimetry (DSC) –* Thermal analysis using a DSC method was carried out employing an automatic thermal analyzer system (Mettler 821^e^). The data processing system (Mettler 821^e^/500/847 thermo-cryostat) was connected to the thermal analyzer. Sealed and holed aluminum pans were used for the experiment for all the samples. Temperature calibrations were made using indium as standard. An empty pan, sealed in the same way as the sample, was used as reference. The thermograms were run at a scanning rate of 10 ºC/min, from 30 to 320 ºC. In many cases it was rather difficult to clearly assess the onset of the thermal event due to the irregular shape of the associated endotherm, therefore in this paper each thermal event is indicated with the peak temperature (Tpeak).

*Thermogravimetric analysis (TGA) –* Thermogravimetric analysis was performed with a Mettler Toledo automatic thermal analyzer system TGA/SDTA851^e^/SF/1100). Open alumina crucibles were used for analysis in the temperature range 30–300 ºC at 10 ºC/min scanning rate under nitrogen stream.

*Thermomicroscopy(HSM)* – The thermomicroscope (HSM) contains a heating apparatus (scanning rate: 10 ºC/min (Mettler –Toledo S.p.a., Novate Milanese, Italy) mounted on a microscope (Nikon UN2-PSE100; Nital S.p.a., Florence, Italy). Photos were captured by a Nikon DN100 digital photocamera. 

*Μicro-Raman spectroscopy –* Raman spectra were recorded by means of a Renishaw Raman Invia configured with a Leica DMLM microscope (spatial resolution 1–60 μm^2^), a notch filter to cut off Rayleigh scattering, a monochromator (1800 lines/mm) and a Charge-Coupled Device (CCD) thermoelectrically cooled (203 K) detector. The light sources available were an Ar^+^ laser (514.5 nm) and a diode-laser (780.0 nm). Experimental details: Ar^+^ laser, (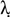 = 514.5 nm), time of each scan: 20 s, number of scans: 4, P_out_ laser: 1.5 mW. 

*Micro-FTIR –* FT-IR (ATR, near-normal reflection-absorption) spectra were recorded by a Nicolet FT-IR Nexus 470 connected to a Nicolet Continuum microscope: Experimental details: source globar (SiC candle); beam splitter m-IR: KBr; detector: MCT (CdTe, doped by Hg) (Hg/Cd); spectral window: 4000–650 cm^-1^; side resolution: 7–80 μm; spectral resolution: 4 cm^-1^*.*

## 4. Conclusions

Diclofenac and its salts with alkyl hydroxy amines are stabilized by a number of hydrogen bonds in the solid state, in such a way that the anion and cation exist as ion pairs. Since in the salts considered here the diclofenac anion is a constant, differences of the solid state can be attributed to the water molecules of crystallization (when present) and/or the hydroxy groups of the cation. 

Taking as a reference the behavior of the salt with TEA, it is possible to observe that three hydroxy groups are necessary to originate only one form in the solid state, without polymorphism and formation of hydrates; the same is encountered for the TRIS salt. Two hydroxy groups, as in the cation of the DEA salt, are sufficient to prevent the formation of hydrates: but the ion pair is kept not so close to prevent the formation of polymorphs. However, the two forms are very similar, as suggested by the only slightly different melting points. When the cation possesses only one hydroxy group (see the MEA salt) the salt needs an additional water molecule of crystallization to fulfill the diclofenac anion requirement of hydrogen bonds: the MEA salt forms a mono-hydrate and, when water is lost by de-hydration, the salt, no longer stabilized by the hydrogen bonds, undergoes polymorph transition.

Substitution of H atoms (linked to the N atom in the bases) with alkyl groups modify the H-bond network in the final salts, that melt at lower temperatures with respect to those formed with unsubstituted hydroxy bases. With the exception of the diMeDEA salt, all the diclofenac salts prepared with these alkyl hydroxy bases exhibit polymorphism.

Hydrophilic groups present in the cations operate to depress the solubility of these salts since they originate a close association in the solid state that may be retained in aqueous solution, but they do not participate in promoting solute/solvent interaction. Formation of hydrates is rarely encountered among these diclofenac salts.
